# Exploring Free Energy Profiles of Enantioselective Organocatalytic Aldol Reactions under Full Solvent Influence

**DOI:** 10.3390/molecules25245861

**Published:** 2020-12-11

**Authors:** Moritz Weiß, Martin Brehm

**Affiliations:** Institut für Chemie, Martin-Luther-Universität Halle-Wittenberg, von-Danckelmann-Platz 4, D-06120 Halle (Saale), Germany; moritz.weiss@chemie.uni-halle.de

**Keywords:** aldol reaction, organocatalysis, enantioselectivity, free energy profile, metadynamics, molecular dynamics, ab initio molecular dynamics, nudged elastic band, energy barrier, solvent influence

## Abstract

We present a computational study on the enantioselectivity of organocatalytic proline-catalyzed aldol reactions between aldehydes in dimethylformamide (DMF). To explore the free energy surface of the reaction, we apply two-dimensional metadynamics on top of *ab initio* molecular dynamics (AIMD) simulations with explicit solvent description on the DFT level of theory. We avoid unwanted side reactions by utilizing our newly developed hybrid AIMD (HyAIMD) simulation scheme, which adds a simple force field to the AIMD simulation to prevent unwanted bond breaking and formation. Our condensed phase simulation results are able to nicely reproduce the experimental findings, including the main stereoisomer that is formed, and give a correct qualitative prediction of the change in *syn:anti* product ratio with different substituents. Furthermore, we give a microscopic explanation for the selectivity. We show that both the explicit description of the solvent and the inclusion of entropic effects are vital to a good outcome—metadynamics simulations in vacuum and static nudged elastic band (NEB) calculations yield significantly worse predictions when compared to the experiment. The approach described here can be applied to a plethora of other enantioselective or organocatalytic reactions, enabling us to tune the catalyst or determine the solvent with the highest stereoselectivity.

## 1. Introduction

One of the most important tasks in organic chemistry is the controlled formation of new C–C bonds in order to build up complicated molecules from simpler educts. Particular attention is paid to the case when a new stereocenter is created by the bond formation: it is well known that different enantiomers or diastereomers of the same compound behave vastly different in many applications (just consider, e.g., drugs). Therefore, it is highly desirable to control the stereochemistry of the product when the formation of the C–C bond creates a new stereocenter. The field of chemistry concerned with this question is called enantioselective synthesis. These methods are highly relevant far beyond academic use—for instance, the chiral drug industry has become an incredibly fast-growing segment of the drug market [1].

One of the most efficient ways to introduce defined chirality into a molecule is to use a catalytic amount of a chiral controller to induce the stereoselective transformation. Those controllers often were organometallic compounds earlier, but with the emergence of the concepts of green chemistry [2,3,4,5,6,7,8,9], also metal-free organic molecules were found to serve this purpose, and the way to the field of organocatalysis was opened. Organocatalysis refers to a form of catalysis where the rate of a chemical reaction is increased by an organic catalyst consisting of only non-metal elements [10,11,12]. The use of catalysts which do not contain metals offers several advantages; for example, there is no need to remove traces of potentially toxic metals from the products. When the organocatalyst is chiral, a way towards enantioselective organocatalysis is opened. One of the first chiral organocatalysts applied was the amino acid proline, which can be considered a key step towards green and sustainable enantioselective synthesis [4,5,6,7,8,9,13]. One of the pioneering reactions on that field was the Hajos–Parrish–Eder–Sauer–Wiechert reaction [14] which has been developed in the 1970s and applies naturally occurring chiral proline in an aldol reaction. Other prominent examples are the asymmetric synthesis of the Wieland–Miescher ketone [15] which is also based on proline, or one transformation in the total synthesis of erythromycin by Robert B. Woodward [16,17,18].

A very popular transformation in organic chemistry is the aldol reaction, which has been discovered independently by the Russian chemist Alexander Borodin in 1869 [19] and by the French chemist Charles-Adolphe Wurtz in 1872 [20]. Within the scope of the reaction, two carbonyl compounds are connected by the formation of a C–C bond, and a β-hydroxy carbonyl compound (also known as aldol) is obtained as the product. The product of aldol reactions can sometimes lose a molecule of water to form an α,β-unsaturated carbonyl compound; then, the reaction is often termed as aldol condensation instead. A variety of different nucleophiles may be employed in the aldol reaction, including enols, enolates as well as enol ethers of ketones, aldehydes, and many other carbonyl compounds. The electrophilic partner is usually an aldehyde or ketone (but there exist many variations, such as the Mannich reaction).

In the generic case, two new stereocenters are formed, and therefore, four different product configurations can be obtained. While the original aldol reaction does not possess any stereoselectivity, modern variants of the aldol reactions allow for controlling both the relative and absolute configuration of the product, while achieving high yields at the same time [21]. Stereogenic aldol units are, e.g., common in polyketides, which possess potent biological properties: the immunosuppressant FK506 [22], the anti-tumor agent discodermolide [23], and the antifungal agent amphotericin B [24] are three examples of such compounds with chiral aldol units. While earlier the synthesis of many such compounds was considered nearly impossible, modern aldol methodology has allowed for their efficient synthesis in many cases [25], supported for example by the application of NMR spectroscopy to understand mechanistic details [26,27,28]. One of the first organocatalysts for stereoselective aldol reactions was proline, as reported in the 1970s by groups at Schering AG and Hoffmann–La Roche [29,30,31]. Despite having been found almost 50 years ago, proline catalysis is still widely applied in enantioselective synthesis in order to preferably form a specific enantiomer or diastereomer in aldol reactions [29,32,33,34].

**Figure 1:**
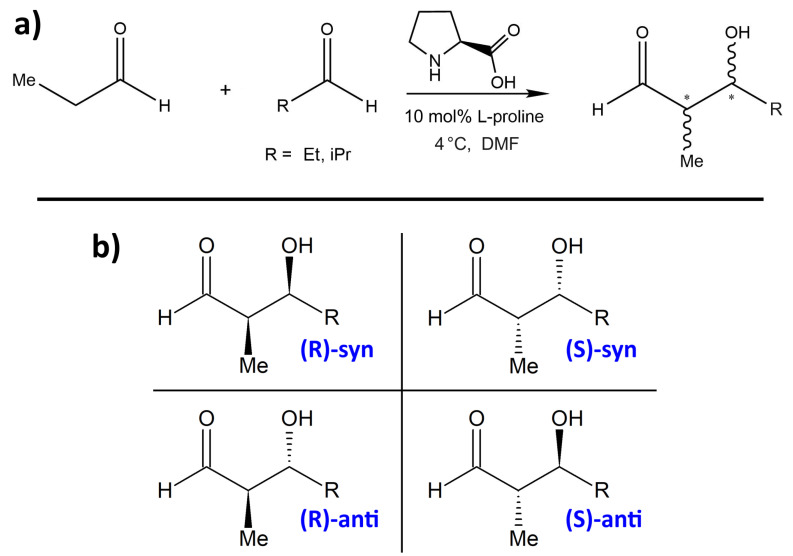
(**a**) The proline-catalyzed enantioselective aldol reaction studied in this work, see [33]. Selectivity and yield are given in Table 1. * denotes the two newly created stereocenters. (**b**) The four possible reaction products of the aldol reaction between two aldehydes. With proline catalysis, the preferred product is always the *(S)-anti* configuration (lower-right corner) [33].

One proline-catalyzed aldol reaction with particularly high enantioselectivity over a wide range of substrates has been experimentally studied by Northrup et al. in 2002 [33]. In particular, this was the first enantioselective catalytic direct cross-aldol reaction that employs nonequivalent aldehydes. The schematic reaction is presented in Figure 1a, and the four possible products are shown in Figure 1b. Without addition of proline as organocatalyst, these four products would be formed in an approximately equal ratio. Northrup et al. report outstanding selectivities due to the catalytic influence of proline. They observed very high % ee values (enantiomeric excess) in the range of 99% for most substituents R, while the *syn:anti* selectivity depends significantly on the choice of R and is found to be between 1:3 and 1:24. The preferred product is always the *(S)-anti* form (see lower-right corner in Figure 1b). When using dimethylformamide (DMF) as the solvent, the total conversion is above 90%. Syringe pump addition of the donor aldehyde to the acceptor aldehyde in the presence of the catalyst effectively suppressed homodimerization of the donor aldehyde. Among all the choices of substituents R in the article, we only consider R = Et, iPr here. The experimentally determined selectivities and yields from [33] for these two substituents are given in Table 1.

**Figure 2:**
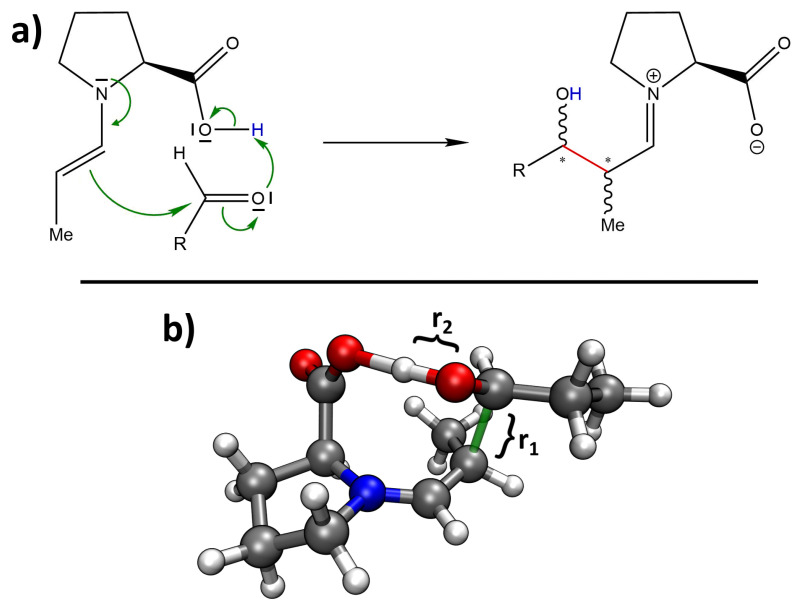
(**a**) The mechanism of the selectivity-determining step of the proline-catalyzed aldol reaction as known from textbooks. A new C–C bond is formed (red), and a proton is shifted (blue). * denotes the two newly created stereocenters. (**b**) Snapshot of the computed transition state of the reaction which leads to the *(S)-anti* product. The newly formed C–C bond is shown in green. The two distances $r_{1}$ and $r_{2}$ are used as collective variables to explore the free energy profile in the second part.

The proline-catalyzed aldol reaction takes place in several steps. First, the proline attacks the carbonyl carbon atom of the donor aldehyde with its nucleophilic nitrogen atom, forming an amino alcohol. In a second step, this amino alcohol loses a water molecule, forming an enamine which is in tautomeric equilibrium with a corresponding iminium ion. In the third step (which is decisive for stereoselectivity), the double bond of the enamine attacks the carbonyl carbon atom of the acceptor aldehyde, forming the C–C bond. Finally, the catalytic proline molecule is cleaved via the addition of water and re-formation of the original aldehyde group of the donor aldehyde. The mechanism of the third step (as known from organic chemistry textbooks) is depicted in Figure 2a. As this is the sole step that determines the stereochemistry of the product, we focus on this step here. Figure 2b shows a 3D rendering of the computed transition state towards the preferred *(S)-anti* product; the newly formed C–C bond is shown in green.

A lot of effort has been undertaken to computationally describe and understand the selectivity of organic reactions. Reactions already studied in literature include the Wittig reaction [35], organocatalytic addition to α,β-unsaturated ketones [36], chiral catalyzed ketimine-ene reactions [37], weak acid dissociation [38], organocatalytic S${}_{N}$2 reactions [39,40], the catalytic vinylogous Henry reaction [41], base-catalyzed Knoevenagel condensations [42,43], the Meyer–Schuster rearrangement [44], enantiomerization of axially chiral biphenyls [45], and also proline-catalyzed reactions [46,47,48]. While some of these studies obtained their results by using static quantum chemical calculations and frequently described solvent influence via continuum solvation models [37,39,40,42,43,44,49,50,51], others were based on molecular dynamics simulations and often used tools such as metadynamics [35,38,43,46,52,53,54] and umbrella sampling [45] to investigate the potential energy surfaces of the reactions. The metadynamics approach, originally proposed by Parrinello et al. [55], reveals the free energy surface of one or more reaction coordinates by applying an adaptive bias potential. It allows us to include the full explicit solvent effect and all entropic terms at the selected temperature, which is in contrast to static calculations, which only include solvents via implicit continuum solvent models, and only capture a rough estimate on entropic contributions via harmonic frequency calculations.

In this article, we perform a computational investigation of the proline-catalyzed enantioselective aldol reaction between aldehydes in DMF, as it has been experimentally studied by Northrup et al. [33]. To explain this high enantioselectivity, we use *ab initio* molecular dynamics (AIMD) in conjunction with metadynamics to explore the two-dimensional free energy surface of the reaction, including the full explicit solvent influence as well as full entropic contributions at room temperature. We avoid unwanted side-reactions by employing our newly developed simulation approach of hybrid AIMD simulation. This is the first time that the free energy profile of an aldol reaction in an explicit organic solvent is presented and discussed in the literature. Our computational results agree nicely with the experimental findings. Based on our computed free energy profiles, we give a microscopic explanation for the high selectivity.

The article is structured as follows. After a description and validation of our newly developed hybrid AIMD simulation approach, we present and discuss the results from static NEB calculations, metadynamics simulations in vacuum, and metadynamics simulations in explicit solvent, respectively. Finally, we investigate the change in selectivity when going to a different substrate molecule. Subsequently, we describe our computational methods. Our manuscript ends with conclusions and an outlook.

## 2. Results and Discussion

### 2.1. Hybrid Simulation Approach

When performing metadynamics with *ab initio* molecular dynamics (AIMD) simulations in order to study free energy profiles of chemical reactions, the forced bond-breaking leads to all kinds of unwanted side reactions in the simulations: the transition states of reactions often feature highly reactive sites (radicals, carbenes, carbocations, etc.), which readily react with the closest solvent molecule to form adducts. From a fundamental view, this is not a problem for metadynamics, because these adducts will be cleaved again later in the simulation, as it also happens in reality. However, simulation time is spent on regions of the potential energy surface which are not relevant for the formation of the desired reaction product. The metadynamics would be required to run for a very long simulation time in order to reach convergence. It would be desirable to have an approach which somehow blocks all unwanted side reactions (i.e., breaking bonds which should remain intact or forming bonds where it is not desired), so that only the regions of the potential energy surface which are relevant for the formation of the main reaction product are sampled and explored. This is only possible if the mechanism of the reaction is known *a priori*—however, this needs to be the case anyway in a metadynamics simulation, because the choice of collective variables needs to match the presumed mechanism.

To do so, we have developed the so-called hybrid AIMD simulation approach (in short “HyAIMD”), which we present for the first time in this article. The foundation of our approach is a standard AIMD simulation (in our case, a Born–Oppenheimer MD simulation). On top of that, a simple and automatically generated force field is applied, and the potential energy and force contribution from the force field is simply added to those of the AIMD. The force field parameters are determined in the following way: all covalent bonds in the system which exist in the initial configuration and shall remain intact during the simulation are assigned a quadratic bond stretching term of the form

(1) $$E^{\mathrm{bond}}\left(r\right):=\frac{k}{2}{\left(r-r_{0}\right)}^{2}$$

with a force constant of $k=4.184\times 10^{-2}\;\mathrm{kJ}\;{\mathrm{mol}}^{-1}\;{\mathrm{pm}}^{-2}$. The equilibrium bond length $r_{0}$ for each bond is determined as the average bond length of that specific bond in a short standard AIMD run without the additional force field. In addition to that, all atoms are assigned Lennard-Jones parameters σ and ϵ, and a non-bonded potential energy contribution $E_{ij}^{\mathrm{LJ}}\left(r\right)$ for all pairs of atoms i,j in a distance *r* is computed as

(2) $$E_{ij}^{\mathrm{LJ}}\left(r\right):=4\epsilon_{ij}\left[{\left(\frac{r}{\sigma_{ij}}\right)}^{12}-{\left(\frac{r}{\sigma_{ij}}\right)}^{6}\right]$$

where the Lennard-Jones cross terms are computed according to the so-called arithmetic mixing rules

(3) $$\sigma_{ij}:=\frac{1}{2}\left(\sigma_{i}+\sigma_{j}\right),\;\epsilon_{ij}:=\sqrt{\epsilon_{i}\;\cdot \;\epsilon_{j}}$$

This pair-wise interaction is only computed for pairs of atoms which shall not form a covalent bond during the simulation; all pairs which shall be allowed to form a bond are excluded. Likewise, all 1–2, 1–3, and 1–4 neighbors (in terms of covalent bonds existing in the beginning) are excluded. A value of $\epsilon_{i}=0.5\;\mathrm{kJ}\;{\mathrm{mol}}^{-1}$ is set for all atoms (no matter of the element). This relatively small value ensures that there is almost no artificial attraction, and only the repulsive part of the potential is relevant. The $\sigma_{i}$ values are set to two times the covalent radii of the corresponding elements (taken from literature [56]), except for hydrogen, where the multiplication by two is omitted, and just the covalent radius is utilized. No partial charges are assigned to the atoms, and no electrostatic interactions are computed in the force field part.

We claim that our hybrid AIMD simulation technique does not significantly alter the structure and dynamics of condensed phase simulations as long as no (unwanted) chemical reactions occur. To prove this claim, we utilized the CP2k program package [57,58] to perform a HyAIMD simulation of the ionic liquid 1-ethyl-3-methylimidazolium acetate ([EMIm][OAc]) in the liquid phase as an example. As standard AIMD simulation trajectories of this system already exist (see literature [59,60,61]), we were able to monitor the changes in structure and dynamics introduced by the addition of the simple force field.

**Figure 3:**
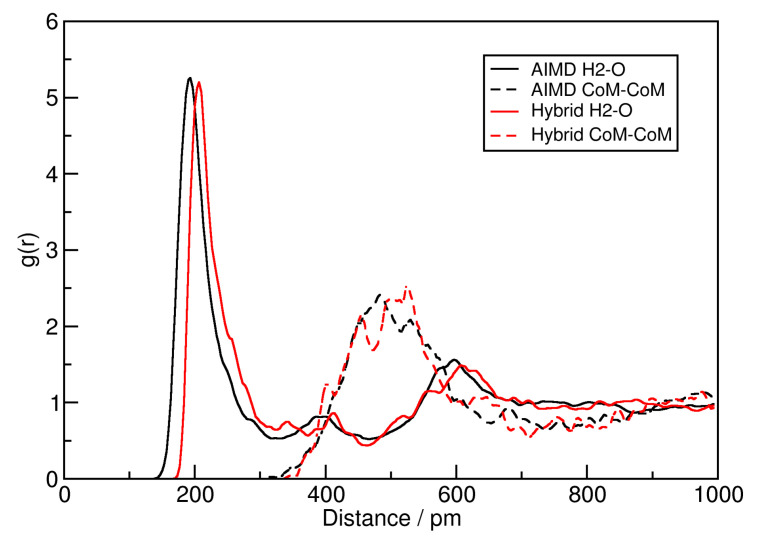
Radial distribution functions (RDFs) between [EMIm]${}^{+}$ H2 and [OAc]${}^{-}$ O (solid lines) and between cation and anion mass centers (dashed lines) of the ionic liquid [EMIm][OAc] from the standard *ab initio* molecular dynamics (AIMD) simulations (black lines) [59,60,61] and the HyAIMD simulations performed here (red lines).

To investigate changes in the liquid structure, two radial distribution functions (RDFs) are shown in Figure 3. While the solid lines correspond to the hydrogen bond between the central ring hydrogen atom H2 of [EMIm]${}^{+}$ and acetate oxygen (for atom nomenclature, see Ref. [59]), the dashed lines depict the center of mass distance between [EMIm]${}^{+}$ cations and acetate anions. The black curves are computed from the original AIMD simulations, while the red curves stem from the HyAIMD trajectory computed here. From both RDFs, it can be clearly seen that the liquid structure and hydrogen bond geometry is not significantly altered by the additional force field terms. The average hydrogen bond length is shifted to slightly larger values (approx. 10 pm), which is expected due to the additional Lennard-Jones repulsion between hydrogen and oxygen atoms, but this should not have a significant influence on the overall properties.

**Figure 4:**
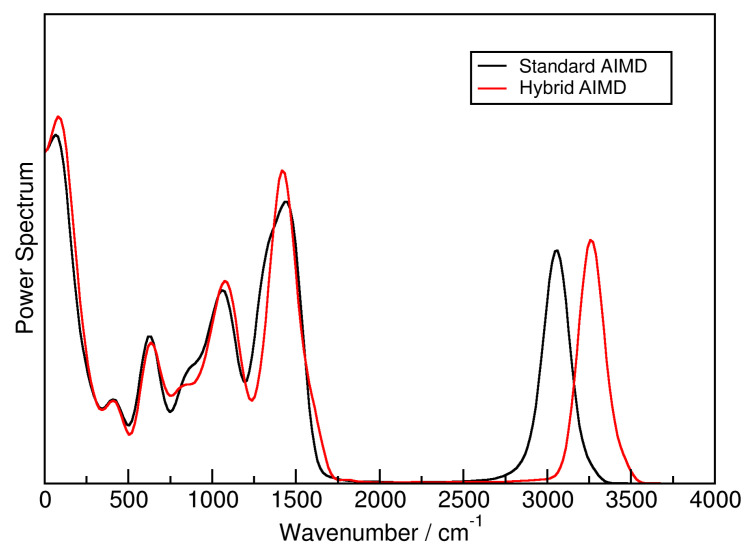
Power spectra of of the ionic liquid [EMIm][OAc], computed from the standard AIMD simulations (black line) [59,60,61] and the HyAIMD simulations performed here (red line).

In order to determine the influence of the HyAIMD approach on the short-time dynamics of the system, the power spectra of the standard AIMD (black line) and the HyAIMD simulation (red line) are presented in Figure 4. It is visible that the vibrational stretching motion of the C–H bonds in the system is shifted to higher wavenumbers by approximately 200 cm${}^{-1}$ when the force field is added. We consider this as not critical for the estimation of reaction free energy profiles, because atom pairs which take part in the reaction (e.g., in the proton transfer) are not influenced by the force field. All the other vibrations, on the other hand, are almost invariant under this change (including the fingerprint region, where deformations of the molecular backbone take place).

Based on these observations, we conclude that our HyAIMD approach leaves the aspects of the system’s structure and dynamics invariant which are relevant for the estimation of free energy profiles of chemical reactions, while it avoids all unwanted side reactions (e.g., with the solvent). Therefore, it should make metadynamic simulations of such reactions much easier, and significantly reduce the required computer time.

### 2.2. Static Nudged Elastic Band Calculations

The first part of this section will focus on the case of R = Et, i.e., a symmetric aldol reaction between two molecules of propionaldehyde. In the first step of our computational investigation, we have performed static nudged elastic band (NEB) calculations of the reaction step which determines the stereochemistry of the product, see Figure 2a. This approach has been used many times in literature to compute the energy barrier of reactions [62,63,64,65,66,67]. Based on optimized structures of the educts and the product, a minimal energy path between these two is constructed. It is not possible to include explicit solvent effects in this approach. There is the option to employ implicit continuum solvent models, but we decided to not do so, because we will investigate the explicit solvent effect later in this manuscript. As this is a static method, it does not include any entropic effects. Simple approximations (particle in a box/rigid rotator/harmonic oscillator) can be employed to estimate the entropy of the educts and product and of the transition state. For the sake of completeness, we have performed this estimation as implemented in ORCA, and we observe below that this did not improve the prediction in any way.

**Figure 5:**
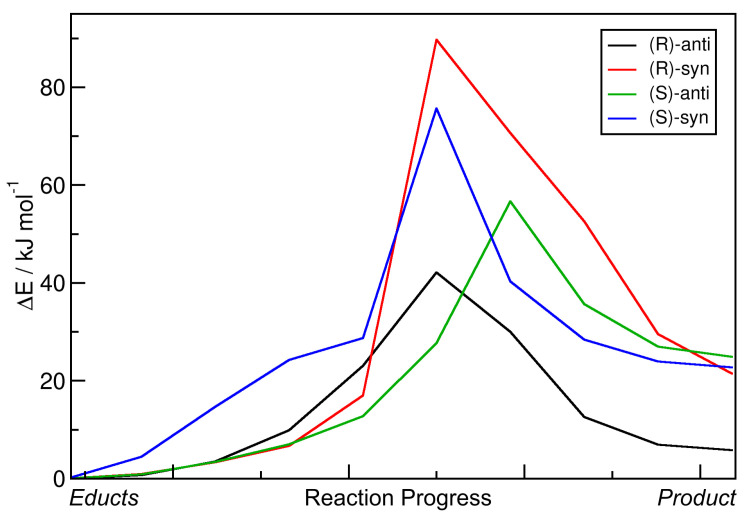
Energy profiles of aldol reactions (R = Et) resulting from static nudged elastic band (NEB) calculations. Energies are given relative to educts.

The resulting energy profiles of the static NEB calculations for the aldol reactions with R = Et are presented in Figure 5. All energies are given relative to those of the educts. The horizontal axis depicts the reaction coordinate which leads from the educts (left) to the products (right). For the four possible products and the nomenclature, please see Figure 1b. It can be seen that all reaction energies $\Delta_{R}$E are positive, i.e., all reactions are endothermic in this approximation. The reaction energy barriers ΔE${}^{‡}$ are found to be in a range of 40–80 kJ mol${}^{-1}$. An overview over these numbers is given in Table 2. Note that the transition state energies ΔE${}^{‡}$ given in the table have been obtained by transition state optimizations subsequent to the NEB calculations. Therefore, the numbers can be slightly different from the maximum of the curves in the diagram. Based on harmonic frequency calculations, we estimated the free reaction enthalpies $\Delta_{R}$G and the free enthalpy barriers ΔG${}^{‡}$ at 300 K as implemented in ORCA; they are also reported in Table 2.

Based on these data, the reaction leading to the formation of the *(R)-anti* configuration should be dominant—it features the lowest energy barrier and is the least endothermic by far among the four reactions. However, in reality, the *(S)-anti* product is dominantly formed (with an %ee value of ≈99%). We conclude that these static NEB calculations are not suitable to reproduce and understand the experimentally found enantioselectivity, not even to predict the main product. The only aspect which is correctly reproduced here is that the two *anti* products are preferred in terms of barrier height (i.e., lower barrier), as it is also the case in experiment (see Table 1). When considering the free enthalpies ΔG instead, all trends remain the same, and the reactions are now even more unfavorable, with free reaction enthalpies in the range of 25–40 kJ mol${}^{-1}$. This can be easily understood from the fact that two molecules react to one molecule here, hence reducing the entropy. Including entropic effects via a simple approximation, therefore, did not improve the results at all.

### 2.3. Metadynamics Simulations in Vacuum

In order to capture the effect of entropy and finite temperature in our predictions (beyond the crude approximation via harmonic frequencies), we performed two-dimensional metadynamics simulations on top of our new HyAIMD simulation approach to explore the free energy profile of the reaction step shown in Figure 2a. The two collective variables which span our configuration space to explore were chosen to be the C–C distance of the newly formed bond and the O–H distance in the alcohol group of the product. These two distances are visualized as $r_{1}$ and $r_{2}$ in Figure 2b. In principle, metadynamics is able to predict the free energy profile ΔF of a configuration space, where ΔF=ΔE−T·ΔS includes the entropic contribution ΔS at the simulation temperature *T*, which was set to 300 K in our case. The simulations presented here were performed in vacuum (technically, the simulation cells contained some noble gas atoms to improve ergodicity; see Section 3.3 below). Metadynamics also allows to include explicit solvent; this will be investigated in Section 2.4.

**Figure 6:**
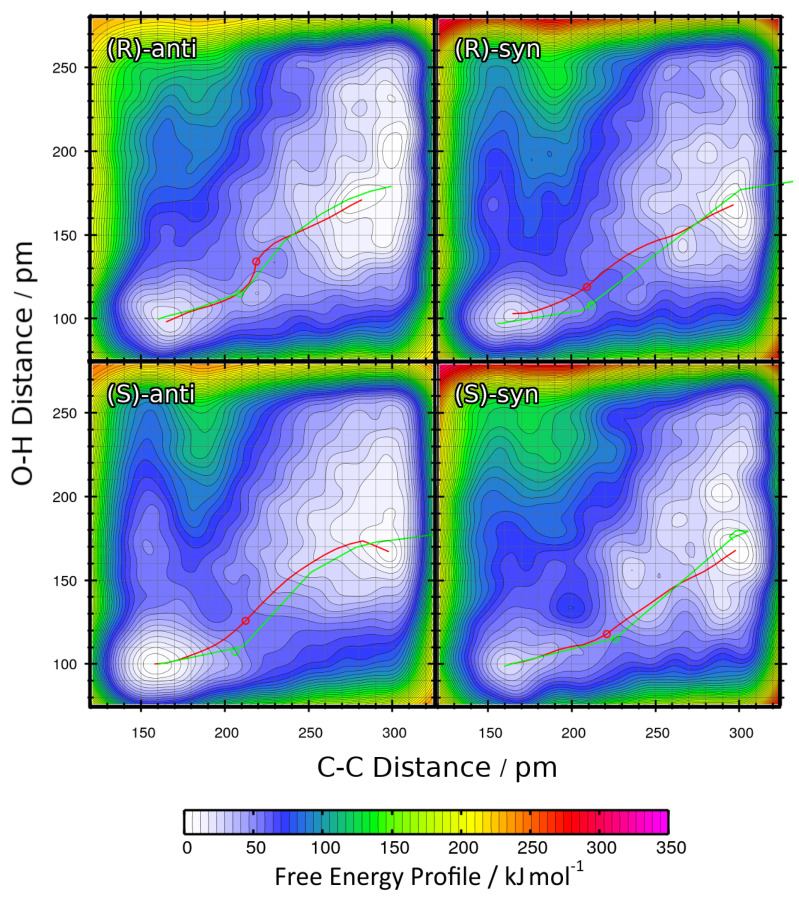
Free energy profiles of the aldol reactions (R = Et) in vacuum computed from HyAIMD metadynamics; definition of collective variables see Figure 2b. Red curves depict the minimum energy paths from educts (upper-right energy basin) to products (lower-left energy basin). Green curves show the results of static NEB calculations for comparison. Green circles denote the statically determined transition state.

The resulting two-dimensional free energy profiles for the reactions with R = Et leading to the four possible products (see Figure 1b ) are presented as contour plots in Figure 6. The free energy basins on the lower-left side of each contour plot represent the product state (both C–C and O–H bond intact), while the basins on the upper-right side of each plot correspond to the educts (both bonds broken). Note that the free energies in these plots are not normalized to the educts as in the one-dimensional plots, but rather to the smallest encountered free energy value. Depending on the reaction, this could be either the educt state or the product state. The red paths through these surfaces depict the minimal free energy pathways which connect the educt state with the product state (i.e., one likely path the reaction might take). The green paths are the results of the static NEB calculations from Section 2.2; these are “non-free” minimal energy pathways without entropic contributions and are just shown for reference here. The green circles denote the statically optimized transition states.

**Figure 7:**
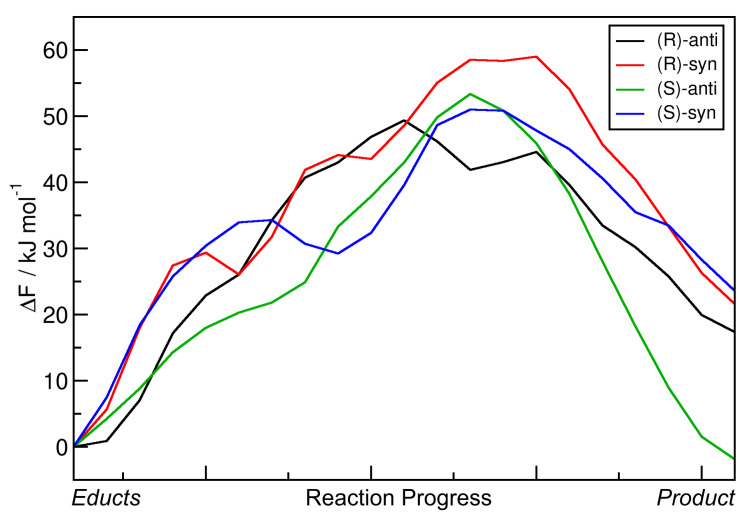
One-dimensional free energy profiles of aldol reactions (R = Et) in vacuum obtained from HyAIMD metadynamics; corresponds to red curves in Figure 6. Free energies are given relative to educts.

For a better comparison, the minimal free energy pathways from Figure 6 (the red paths) have been extracted and are shown in Figure 7 as one-dimensional plots. Note that in the latter plot, the free energies are given relative to the educts, which was not the case in the contour plot. The reaction free energies $\Delta_{R}$F and reaction free energy barriers ΔF${}^{‡}$ are also presented in Table 3.

When considering these data, it becomes clear that the reaction leading to the *(S)-anti* product is the only among the four with negative $\Delta_{R}$F value, i.e., the only exergonic reaction which is predicted to run spontaneously within the scope of this approximation. The other three reactions have reaction free energies in the order of 20 kJ mol${}^{-1}$, and are therefore predicted to not run spontaneously. This is well in line with the experimental results, which state that *(S)-anti* is the observed main product. As in the results from the static NEB calculations (Table 2) the reaction energy of the reaction leading to the *(S)-anti* product was significantly positive, we can conclude that the entropic effects which have been taken into account now favor the formation of the *(S)-anti* product, while this is not the case for the other three products. The free energy barriers ΔF${}^{‡}$ are all in a similar range of ≈50 kJ mol${}^{-1}$, with the exception of the reaction leading to the *(R)-syn* product that possesses a barrier of almost 60 kJ mol${}^{-1}$. This can be correlated to the fact that the *(R)-syn* product possesses both the wrong absolute configuration *(R / S)* and the wrong relative configuration *(syn / anti)* and is therefore doubly unfavorable.

From the free energy profiles in Figure 6, it is well visible that the C–C bond formation and the proton transfer take place in a concerted manner. Doing one step before the other (corresponding to horizontal and vertical paths in the plots) would face very high free energy barriers. The only path which can lead to a successful reaction changes the C–C and O–H distance simultaneously. We would like to point out that this is a result of our study—the choice of collective variables as well as the restraints imposed against side reactions allow for both a concerted and a two-step mechanism. For such a concerted reaction to take place, the two oxygen atoms between which the proton is transferred need to be located at a suitable distance before the reaction starts, so that the proton can be “handed over” from one oxygen atom to the other. Depending on the molecular orientation of the educts before the reaction, this is only possible in some of the geometries. Therefore, the reactions leading to certain configurations are favored above the others, explaining the enantioselectivity.

We conclude this section by noting that the free energy profiles from metadynamics simulations in vacuum give a much better prediction for the stereoselectivity of the aldol reaction; they predict the correct main product and suggest that this reaction will run spontaneously. The atomistic reason for the enantioselectivity is the requirement for close oxygen–oxygen contact to make a concerted reaction possible. However, the reaction free energy barriers are still relatively high (≈50 kJ mol${}^{-1}$), so that the reaction would proceed very slowly. This leads us to the conclusion that the main driving force of the reaction is the explicit solvent influence, which was not included in all calculations above.

### 2.4. Metadynamics Simulations in DMF

After having described the free energy profile of the aldol reaction in a reasonable way above, we finally want to capture the explicit effect of the solvent molecules (DMF) on the reaction. To do so, we applied our new HyAIMD approach to perform metadynamics simulations in the condensed phase, using a fully periodic solvent cell. The HyAIMD approach prevents side-reactions with the solvent from happening, which would be inevitable otherwise when the metadynamics leads to bond breaking. The simulation methodology applied here includes both the full explicit solvent influence on the DFT level of theory and the full entropic effect at the simulation temperature of 300 K, and should therefore yield very reliable free energy surfaces for the reaction.

**Figure 8:**
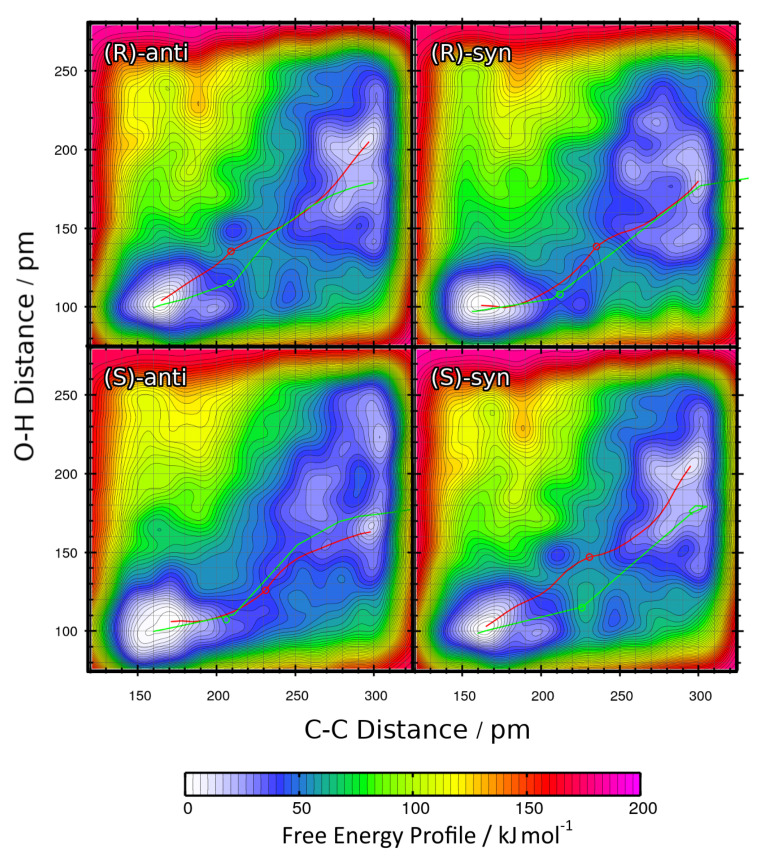
Free energy profiles of the aldol reactions (R = Et) in DMF computed from HyAIMD metadynamics; definition of collective variables see Figure 2b. Red curves depict the minimum energy paths from educts (upper-right energy basin) to products (lower-left energy basin). Green curves show the results of static NEB calculations for comparison. Green circles denote the statically determined transition state.

The two-dimensional free energy profiles obtained from these four metadynamics runs (one per possible product; see Figure 1b ) are shown in Figure 8 as contour plots. Again, the free energy basins on the lower-left side of each plot represent the product state (both C–C and O–H bond intact), while the basins on the upper-right side of each plot correspond to the educts (both bonds broken). As above, please note that the free energies in the contour plots are not normalized to the educts as in the one-dimensional plots, but rather to the smallest encountered free energy value, which is the product here. The red paths through these surfaces depict the minimal free energy pathways which connect the educt state with the product state (i.e., one likely path the reaction might take). The green paths are the results of the static NEB calculations from Section 2.2; these are “non-free” minimal energy pathways without entropic or solvent contributions and are just shown for reference here. The green circles denote the statically optimized transition states.

**Figure 9:**
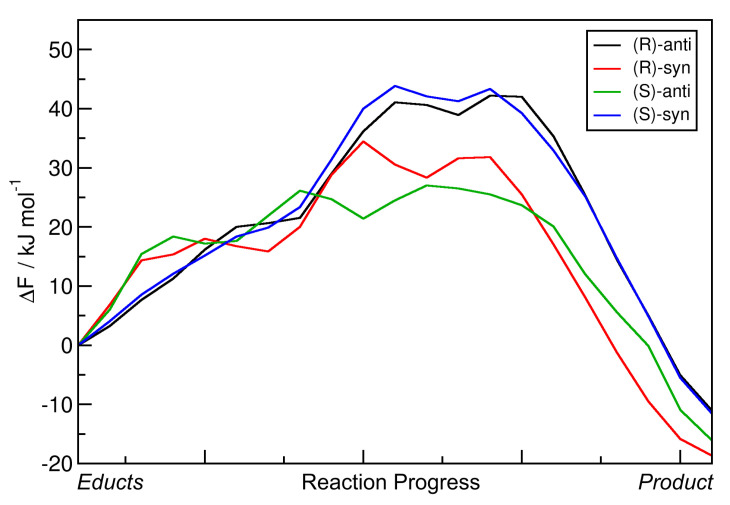
One-dimensional free energy profiles of aldol reactions (R = Et) in dimethylformamide (DMF) obtained from HyAIMD metadynamics; corresponds to red curves in Figure 8. Free energies are given relative to educts.

Very similar to the approach in Section 2.3, the minimal free energy pathways from Figure 8 (the red paths) have again been extracted and are presented in Figure 9 as one-dimensional graphs. The reaction free energies $\Delta_{R}$F and reaction free energy barriers ΔF${}^{‡}$ are also presented in Table 4.

The data show that including the explicit solvent influence brings some significant changes to the free energy surface. Most apparent, all four reactions possess negative reaction free energy values $\Delta_{R}$F now (exergonic), so that all four reactions are predicted to run spontaneously. Both the reactions leading to the *(S)-anti* product (the preferred product) and to the *(R)-syn* product show reaction free energies of around −20 kJ mol${}^{-1}$, while the other two reactions have values of around −10 kJ mol${}^{-1}$ here. The free energy barriers have all been significantly lowered by the addition of solvent. While they have been in the range of 50–60 kJ mol${}^{-1}$ in vacuum (see Table 3), they are now all in a range of 25–40 kJ mol${}^{-1}$. In particular, the free energy barrier leading to the experimentally preferred *(S)-anti* product is only 25 kJ mol${}^{-1}$, which is as weak as a strong hydrogen bond and can be easily overcome by the thermal energy at room temperature.

When compared to the metadynamics simulations in vacuum (see Figure 6), the free energy profiles in Figure 8 possess even higher barriers for non-concerted reaction mechanisms; those would correspond to horizontal and vertical paths in the profile. The only viable path for the reaction is the concerted mechanism, in which the C–C bond formation takes place simultaneously with the proton transfer. The explicit description of the solvent even intensified this effect by making the valley leading from educts to product more narrow.

To conclude this section, we observe that the inclusion of explicit solvent influence significantly lowers the reaction free energies $\Delta_{R}$F; all four reactions are exergonic now. At the same time, it significantly reduces the free energy barrier heights down to values that can be overcome by thermal energy at room temperature. Or in other words, only the presence of explicit solvent allows this reaction to happen at all. The main product observed in the experiment is correctly predicted by our approach, it possesses by far the smallest free energy barrier of only 25 kJ mol${}^{-1}$ here. Therefore, the HyAIMD metadynamics method is able to predict both the correct absolute *(R/S)* and relative *(syn/anti)* stereochemistry of the product. As noted above, the requirement for close oxygen–oxygen contact to allow for a concerted reaction dictates the high enantioselectivity.

### 2.5. Influence of Substituent

After having extensively discussed the proline-catalyzed aldol reaction between two molecules of propionaldehyde (R = Et), we now consider an asymmetric case in which the donor aldehyde is still propionaldehyde, but the acceptor aldehyde is isobutyraldehyde, i.e., R = iPr. With non-identical donor and acceptor aldehydes, there could be eight different products in principle (if donor and acceptor swap), but the experimental setup made sure that this does not happen by using a syringe pump [33], so that we only have to consider the same four products as before, see Figure 1b.

When considering the R = Et case, we found that both the static NEB calculations and the metadynamics simulations in vacuum were not suitable to correctly predict and describe the reaction outcome, and only the metadynamics simulation with explicit solvent was able to yield results which matched the experiment. Therefore, we will only discuss the explicit solvent approach for the R = iPr case. The data from the other two approaches are presented in the Supporting Information in Figures S2, S5, and S9 as well as Tables S2 and S5.

**Figure 10:**
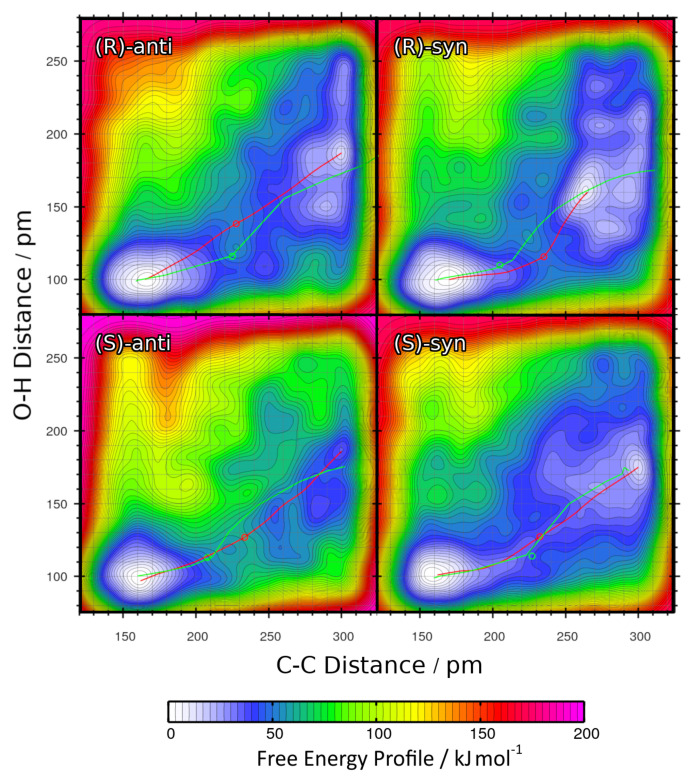
Free energy profiles of the aldol reactions (R = iPr) in DMF computed from HyAIMD metadynamics; definition of collective variables see Figure 2b. Red curves depict the minimum energy paths from educts (upper-right energy basin) to products (lower-left energy basin). Green curves show the results of static NEB calculations for comparison. Green circles denote the statically determined transition state.

The two-dimensional free energy profiles obtained from the four metadynamics runs for R = iPr under the full solvent influence are shown in Figure 10 as contour plots. The free energy basins on the lower-left side of each plot represent the product state (both C–C and O–H bond intact), while the basins on the upper-right side of each plot correspond to the educts (both bonds broken). Note that the free energies in the contour plots are not normalized to the educts as in the one-dimensional plots, but rather to the smallest encountered free energy value, which is the product here. The red paths through these surfaces depict the minimal free energy pathways which connect the educt state with the product state (i.e., one likely path the reaction might take). The green paths are the results of static NEB calculations; they are just shown for reference here. The green circles denote the statically optimized transition states.

**Figure 11:**
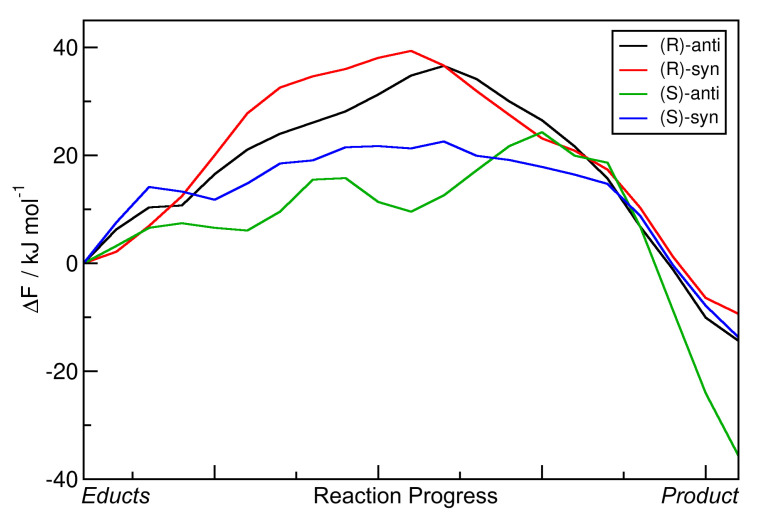
One-dimensional free energy profiles of aldol reactions (R = iPr) in DMF obtained from HyAIMD metadynamics; corresponds to red curves in Figure 10. Free energies are given relative to educts.

As also performed above, the minimal free energy pathways from Figure 10 (the red paths) have been extracted and are presented in Figure 11 as one-dimensional curves. For direct comparison, the reaction free energies $\Delta_{R}$F and reaction free energy barriers ΔF${}^{‡}$ are presented in Table 5.

When comparing this data to those from the R = Et case (Table 4), some notable differences are visible. The reaction free energies $\Delta_{R}$F are now distributed over a wider range of −10 … −35 kJ mol${}^{-1}$, when compared to the span of −10 … −20 kJ mol${}^{-1}$ above. The reaction which leads to the formation of the experimentally favored *(S)-anti* product possesses by far the lowest reaction free energy of −35 kJ mol${}^{-1}$, at least 20 kJ mol${}^{-1}$ smaller than for all the other reactions. The free energy barriers ΔF${}^{‡}$ are found in the range of 20 … 40 kJ mol${}^{-1}$, which is very similar to the R = Et case. The two reactions leading to the *(R)* product (wrong absolute stereochemistry) show significantly larger free energy barriers than the other two reactions. The highest free energy barrier among all four reactions is found when going towards the *(R)-syn* product, which possesses both the wrong absolute and relative stereochemistry.

Another interesting question concerns the *syn:anti* selectivity. The experimental results [33] show that the *syn:anti* ratio in the R = iPr case is 1:24, which is very high when compared to the R = Et case where it was 1:4. It will be interesting to see if this difference in *syn:anti* selectivity between these two substituents is also reproduced in our metadynamics simulations. Indeed, there is a large difference in the relative reaction free enthalpies. In the R = Et case, the difference between $\Delta_{R}$F for *(S)-syn* and *(S)-anti* was 6.19 kJ mol${}^{-1}$. However, in the R = iPr case discussed here, this difference amounts to 21.93 kJ mol${}^{-1}$. This significantly larger free energy advantage for the *(S)-anti* product over the *(S)-syn* product in the R = iPr case can (at least qualitatively) explain the larger *syn:anti* ratio that has been observed in experiment.

We conclude that also for the R = iPr case, our approach is able to correctly describe and predict the stereoselectivity of the organocatalytic aldol reaction. The experimentally observed main product is correctly predicted by a very large reaction free energy gain when compared to the other three products. Even the larger *syn:anti* selectivity when compared to the R = Et case is qualitatively predicted by our computations, which suggests that the effect of the substituent R on the free energy profile is correctly captured by the methodology.

## 3. Methods

Figure 3, Figure 4, Figure 5, Figure 7, Figure 9 and Figure 11 were created with xmgrace [68]. Figure 6, Figure 8 and Figure 10 have been obtained from Gnuplot [69]. Figure 2b was rendered with VMD [70] and Tachyon [71]. Analysis of the simulation trajectories was carried out with TRAVIS [72,73].

### 3.1. Static Calculations and Nudged Elastic Band

The geometry optimizations of the educts and products in vacuum, as well as the static nudged elastic band (NEB) calculations were performed with the ORCA program package [74,75,76], using basis sets of the kind def2–TZVPP [77], RI auxiliary basis sets of the kind def2/J [78], and Grimme’s D3 dispersion correction [79,80] with Becke–Johnson damping. The self-consistent field (SCF) convergence criterion was set to “VeryTightSCF”, and the settings “Grid5 NoFinalGrid” were used for the DFT grid. After the climbing image NEB (CI–NEB) calculations were converged, a subsequent transition state optimization was started from the climbing image, and harmonic frequency calculations were employed to ensure that the optimized transition states possess exactly one imaginary frequency.

### 3.2. Force Field Pre-Equilibration

The simulations of the aldol reactions were started from optimized structures of the respective products. In order to obtain initial configurations with a reasonable orientation of solvent molecules around the substrate, we employed a simple force field pre-equilibration scheme. The optimized structure of the reaction product was placed in the middle of a cubic simulation cell with around 1.4 nm edge length, and all atoms of the product were frozen. By doing so, we did not require bonded interaction parameters for these organic molecules. Atomic partial charges were then computed by the restraint electrostatic potential (RESP) approach [81] as implemented in CP2k [82]. Lennard-Jones parameters for the elements were taken from the OPLS–AA force field [83]. Then, 19 DMF molecules were randomly placed in the simulation cell by using Packmol [84], and an MD simulation of these molecules around the frozen reaction product was carried out with LAMMPS [85] in order to obtain a reasonable solvent orientation. The final snapshots of these simulations were used as starting configuration for the subsequent step.

### 3.3. Ab Initio Molecular Dynamics Simulations

For the BOMD simulations, we used the program package CP2k [57,58], employing the Quickstep method [86] and orbital transformation [87]/DIIS for fast convergence. The electronic structure was calculated with density functional theory [88,89], utilizing the BLYP functional [90,91] together with the recent reparametrization [92] of Grimme’s D3 dispersion correction [79,80] with Becke–Johnson damping. Basis sets of the kind MOLOPT-DZVP-SR-GTH [93] and GTH pseudopotentials [94,95] were applied to all atoms. The plane wave cutoff was set to 350 Ry, and an SCF convergence criterion of $10^{-6}$ was used. The time step was chosen to be 0.5 fs in all simulations. The simulation temperature was adjusted to 300 K by a Nosé–Hoover chain thermostat [96,97,98] (i.e., NVT ensemble). After an equilibration period of 1 ps with massive thermostats and thermostat time constant τ=10fs, a global thermostat with τ=50fs was applied for the production runs and metadynamics simulations. The “vacuum” AIMD simulations contained 17 argon atoms within the periodic simulation cell in order to improve ergodicity by regularly transferring momentum and energy to different parts of the organic molecule.

### 3.4. Hybrid Simulation Approach

In order to avoid unwanted side reactions and to save time by avoiding irrelevant regions of the potential energy surface, we employed our newly developed hybrid AIMD approach, which is described in Section 2.1. We started the simulations from the products; therefore, we added the additional bond potential to all covalent bonds except two: the C–C bond which has to be broken to get back to the educts, and the alcohol O–H bond in order to allow the transfer of the proton back to the proline. The repulsive Lennard-Jones potential was added to all pairs of atoms except 1–2, 1–3, and 1–4 bonded neighbors and the pair of the transferred proton and the proline carboxylic oxygen atom which receives this proton. The CP2k input files for the hybrid AIMD and force field simulations were automatically generated by a not yet publicly available module of the TRAVIS program package [72,73]. This module will be shared upon request and will be put into the public TRAVIS version once it is stable and mature enough.

### 3.5. Metadynamics Simulations

To explore the free energy profile of the aldol reaction, we have chosen two collective variables (distances), which are depicted by the two labels $r_{1}$ and $r_{2}$ in Figure 2b. For both collective variables, we employed harmonic upper walls, at 250 pm for $r_{1}$ and at 300 pm for $r_{2}$, with a spring constant of $k=5.25\;\cdot \;10^{-2}$ kJ mol${}^{-1}$ pm${}^{-2}$ in order to not explore regions of the free energy surface which are far on the educt side—this would waste a lot of computational power. In addition to that, we placed a harmonic restraint on the distance between the two oxygen atoms of the system (target value $r_{0}=266$ pm, spring constant $k=4.184\;\cdot \;10^{-4}$ kJ mol${}^{-1}$ pm${}^{-2}$) in order to keep the two oxygen atoms close enough together to allow for the proton transfer (“O–H⋯O”) required during the reaction.

The metadynamics simulations [55] were performed in the extended Lagrangian scheme as proposed by Iannuzzi et al. [99] and implemented in CP2k [57,58], using a virtual particle mass of 98.0 a.m.u., a scale of 15 pm, and a coupling spring constant of $\lambda =0.525\;E_{h}\;a_{0}^{-2}$ for both collective variables. The temperature of the virtual particle was thermostated to 20 K. New Gaussian hills with a height of 1.31 kJ mol${}^{-1}$ and a variance of 15 pm (for both dimensions) were spawned every 40 simulation steps (i.e., every 20 fs). The total simulation time of the metadynamics was around 250 ps in all cases. Within the last 50 ps of the simulations, the height of the spawned hills was reduced to 0.13 kJ mol${}^{-1}$ in order to smoothen the free energy profile.

The 2D contour plots of the free energy profiles in Figure 6 and Figure 8 were created from the CP2k output by a locally developed script. The free energy paths in these contour plots (red curves) have been obtained by using the nudged elastic band (NEB) approach to these energy surfaces, using a locally developed C++ program which is based on the “cppneb” program from Jaap Kroes.

## 4. Conclusions

In this article, we have presented a computational study on the enantioselectivity of the organocatalytic proline-catalyzed aldol reaction in DMF. To explore the free energy surface of the reaction, we have performed two-dimensional metadynamics on top of *ab initio* molecular dynamics (AIMD) simulations with fully explicit solvent description on the DFT level of theory. To avoid unwanted side-reactions during the metadynamics run, we utilize our newly developed hybrid AIMD (HyAIMD) simulation scheme, which adds a simple force field to the AIMD simulation in order to prevent unwanted bond breaking and formation. This ensures that the metadynamics only explores regions of the potential energy surface which contribute to the product formation, and therefore saves a lot of computer time. Benchmark calculations show that this approach has, apart from preventing unwanted reactivity, no significant influence on the structure and dynamics of liquid phase simulations.

Our *ab initio* simulation results with explicit solvent are able to nicely reproduce the experimental findings, including the main product that is formed, and even give a correct qualitative prediction of the change in *syn:anti* product ratio when considering a different substituent. We show that both the explicit description of the solvent and the inclusion of entropic effects are vital to a good outcome—metadynamic simulations in vacuum and in particular static nudged elastic band (NEB) calculations yield significantly worse predictions when compared to the experiment. By studying the free energy profiles, we give a microscopic explanation for the high enantioselectivity: the reaction can only take place in a concerted manner, where the C–C bond formation and the proton transfer take place simultaneously—particularly with explicit solvent, where the valley for the reaction in the free energy profile is very narrow. This is only possible if the two oxygen atoms between which the proton shall be transferred are close enough during the whole process, which is only the case in educt arrangements that lead to the preferred product.

This is the first time that the free energy profile of an organocatalytic aldol reaction in an explicit organic solvent is presented and discussed in the literature, and also the first time that the solvent-dependent enantioselectivity of such a reaction has been predicted by *ab initio* methods. Our approach can be applied to a plethora of other enantioselective (and non-enantioselective) reactions, and also to many more solvents, including, e.g., ionic liquids. With sufficient computer time, a solvent screening can be performed for a given reaction to find the solvent which leads to the highest enantioselectivity, or an organocatalyst can be fine-tuned for better application-specific performance.

One improvement of the approach presented here is to apply well-tempered metadynamics [100] instead, which makes the detection of convergence of the free energy profiles significantly easier, and does no longer require to manually scale down the hill height in the last part of the simulation. We will certainly apply this method in our ongoing work.

## Figures and Tables

**Table 1:** Experimentally determined enantiomeric excess, *syn:anti* selectivity, and yield of the aldol reaction shown in Figure 1a for R = Et, iPr; taken from Ref. [33].

R	%ee	*syn:anti*	Yield/%
Et	99	1 : 4	80
iPr	>99	1 : 24	82

**Table 2:** Total reaction energies $\Delta_{R}$E and reaction barriers ΔE${}^{‡}$ for the aldol reactions (R = Et) from static NEB calculations; see Figure 5. Free enthalpies $\Delta_{R}$G and ΔG${}^{‡}$ estimated from harmonic frequency calculations at 300 K. All energies are given relative to educts.

Product	$\Delta_{R}$E/J mol${}^{-1}$	$\Delta_{R}$G/kJ mol${}^{-1}$	ΔE${}^{‡}$/kJ mol${}^{-1}$	ΔG${}^{‡}$/kJ mol${}^{-1}$
*(R)-anti*	5.86	23.77	40.79	44.78
*(R)-syn*	21.50	39.60	78.22	88.63
*(S)-anti*	24.89	43.23	51.63	62.91
*(S)-syn*	22.74	36.28	75.67	90.88

**Table 3:** Total reaction free energies $\Delta_{R}$F and reaction free energy barriers ΔF${}^{‡}$ for the aldol reactions (R = Et) in vacuum from HyAIMD metadynamics; extracted from Figure 7. Free energies are given relative to educts.

Product	$\Delta_{R}$F/kJ mol${}^{-1}$	ΔF${}^{‡}$/kJ mol${}^{-1}$
*(R)-anti*	17.33	49.38
*(R)-syn*	21.56	59.01
*(S)-anti*	−1.90	53.36
*(S)-syn*	23.59	50.99

**Table 4:** Total reaction free energies $\Delta_{R}$F and reaction free energy barriers ΔF${}^{‡}$ for the aldol reactions (R = Et) in DMF from HyAIMD metadynamics; extracted from Figure 9. Free energies are given relative to educts.

Product	$\Delta_{R}$F/kJ mol${}^{-1}$	ΔF${}^{‡}$/kJ mol${}^{-1}$
*(R)-anti*	−11.06	42.24
*(R)-syn*	−18.66	31.79
*(S)-anti*	−17.71	25.40
*(S)-syn*	−11.52	43.84

**Table 5:** Total reaction free energies $\Delta_{R}$F and reaction free energy barriers ΔF${}^{‡}$ for the aldol reactions (R = iPr) in DMF from HyAIMD metadynamics; extracted from Figure 11. Free energies are given relative to educts.

Product	$\Delta_{R}$F/kJ mol${}^{-1}$	ΔF${}^{‡}$/kJ mol${}^{-1}$
*(R)-anti*	−14.33	36.54
*(R)-syn*	−9.35	39.35
*(S)-anti*	−35.63	24.29
*(S)-syn*	−13.70	22.59

## Supplementary Materials

The following are available online, Figure S1: Energy profile of aldol reaction in vacuum with R = Et from static NEB calculation, Figure S2: Energy profile of aldol reaction in vacuum with R = iPr from static NEB calculation, Figure S3: Two-dimensional free energy profile of aldol reaction in vacuum with R = Et from metadynamics, Figure S4: Two-dimensional free energy profile of aldol reaction in DMF with R = Et from metadynamics, Figure S5: Two-dimensional free energy profile of aldol reaction in vacuum with R = iPr from metadynamics, Figure S6: Two-dimensional free energy profile of aldol reaction in DMF with R = iPr from metadynamics, Figure S7: One-dimensional free energy profile of aldol reaction in vacuum with R = Et from metadynamics, Figure S8: One-dimensional free energy profile of aldol reaction in DMF with R = Et from metadynamics, Figure S9: One-dimensional free energy profile of aldol reaction in vacuum with R = iPr from metadynamics, Figure S10: One-dimensional free energy profile of aldol reaction in DMF with R = iPr from metadynamics, Table S1: Total reaction energies and energy barriers of aldol reaction in vacuum with R = Et from static NEB calculations, Table S2: Total reaction energies and energy barriers of aldol reaction in vacuum with R = iPr from static NEB calculations, Table S3: Total reaction free energies and free energy barriers of aldol reaction in vacuum with R = Et from metadynamics, Table S4: Total reaction free energies and free energy barriers of aldol reaction in DMF with R = Et from metadynamics, Table S5: Total reaction free energies and free energy barriers of aldol reaction in vacuum with R = iPr from metadynamics, Table S6: Total reaction free energies and free energy barriers of aldol reaction in DMF with R = iPr from metadynamics.

## Abbreviations

The following abbreviations are used in this manuscript:

AIMD	*ab initio* molecular dynamics
BOMD	Born–Oppenheimer molecular dynamics
DFT	Density functional theory
DMF	Dimethylformamide
%ee	Enantiomeric excess
EMIm	1-Ethyl-3-methylimidazolium cation
Et	Ethyl group
HyAIMD	The hybrid AIMD approach presented here
iPr	Isopropyl group
LJ	Lennard-Jones
MD	Molecular dynamics
NEB	Nudged elastic band
OAc	Acetate anion
RDF	Radial distribution function
RESP	Restraint electrostatic potential
SCF	Self-consistent field
TS	Transition state
