# Supplementary material for: Exploring Free Energy Profiles of Enantioselective Organocatalytic Aldol Reactions under Full Solvent Influence
Source: Molecules. 2020 Dec 11;25(24):5861. doi: 10.3390/molecules25245861 (PMC7764805; doi:10.3390/molecules25245861)
Supplement: Supplementary file 1 [file molecules-25-05861-s001.pdf]

Exploring Free Energy Profiles of Enantioselective Organocatalytic Aldol Reactions under Full Solvent Influence

Supporting Information

Moritz Weiß and Martin Brehm*

Institut für Chemie - Theoretische Chemie, Martin-Luther-Universität Halle-Wittenberg, Von-Danckelmann-Platz 4, 06120 Halle (Saale), Germany.

*E-mail: Martin_Brehm@gmx.de

*Website: https://brehm-research.de/

Static Nudged Elastic Band Calculations

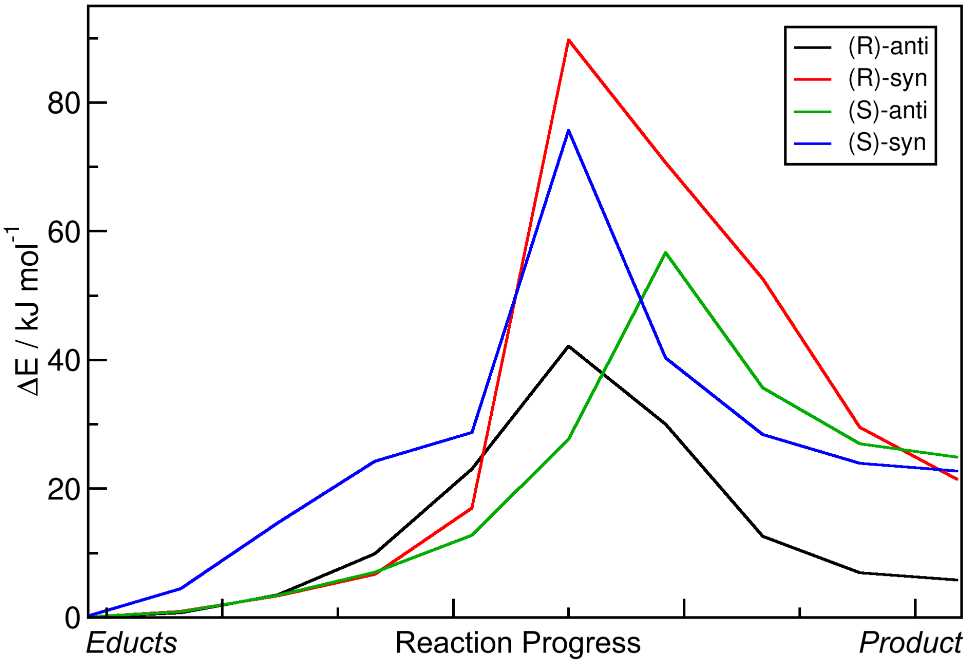

Figure S1: Energy profiles of Aldol reactions (R = Et) resulting from static NEB calculations. Energies are given relative to educts.

Table S1: Total reaction energies $\Delta_R E$ and reaction barriers $\Delta E^{\ddagger}$ for the Aldol reactions (R = Et) from static NEB calculations; see Figure S1. Free enthalpies $\Delta_R G$ and $\Delta G^{\ddagger}$ estimated from harmonic frequency calculations at 300 K. All energies are given relative to educts.

**Product**	Δ_R**E / kJ mol**$^{-1}$	Δ_R**G / kJ mol**$^{-1}$	Δ**E**‡ **/ kJ mol**$^{-1}$	Δ**G**‡ **/ kJ mol**$^{-1}$
(R)-anti	5.86	23.77	40.79	44.78
(R)-syn	21.50	39.60	78.22	88.63
(S)-anti	24.89	43.23	51.63	62.91
(S)-syn	22.74	36.28	75.67	90.88

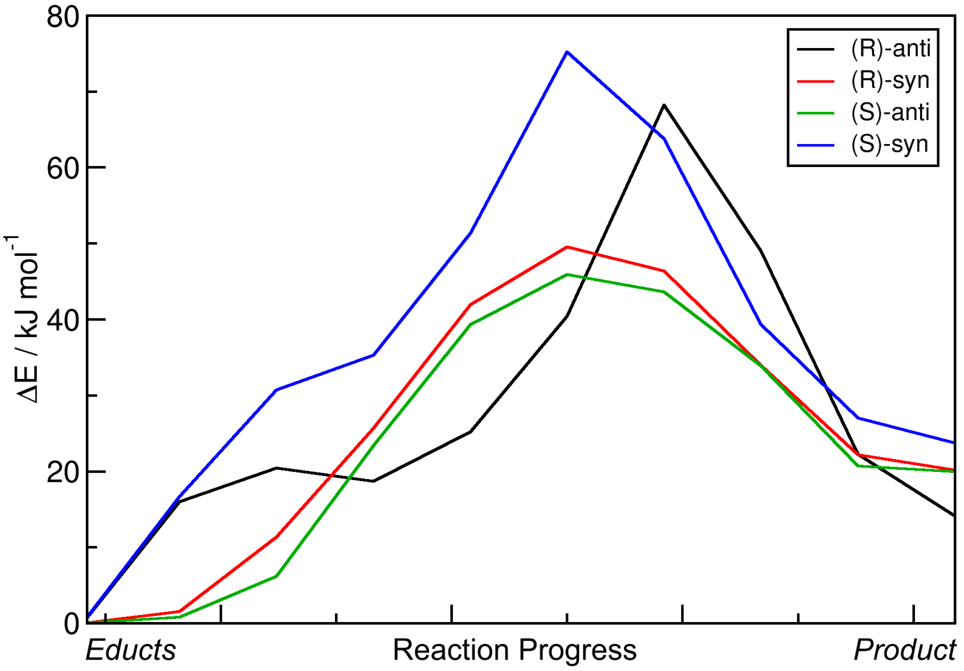

Figure S2: Energy profiles of Aldol reactions (R = iPr) resulting from static NEB calculations. Energies are given relative to educts.

Table S2: Total reaction energies $\Delta_R E$ and reaction barriers $\Delta E^{\ddagger}$ for the Aldol reactions (R = iPr) from static NEB calculations; see Figure S2. Free enthalpies $\Delta_R G$ and $\Delta G^{\ddagger}$ estimated from harmonic frequency calculations at 300 K. All energies are given relative to educts.

**Product**	Δ_R**E / kJ mol**$^{-1}$	Δ_R**G / kJ mol**$^{-1}$	Δ**E**‡ **/ kJ mol**$^{-1}$	Δ**G**‡ **/ kJ mol**$^{-1}$
(R)-anti	14.10	31.29	68.21	73.49
(R)-syn	20.19	38.38	46.68	54.43
(S)-anti	19.95	41.98	42.28	52.15
(S)-syn	23.73	37.25	71.51	73.89

Free Energy Profiles from Metadynamics

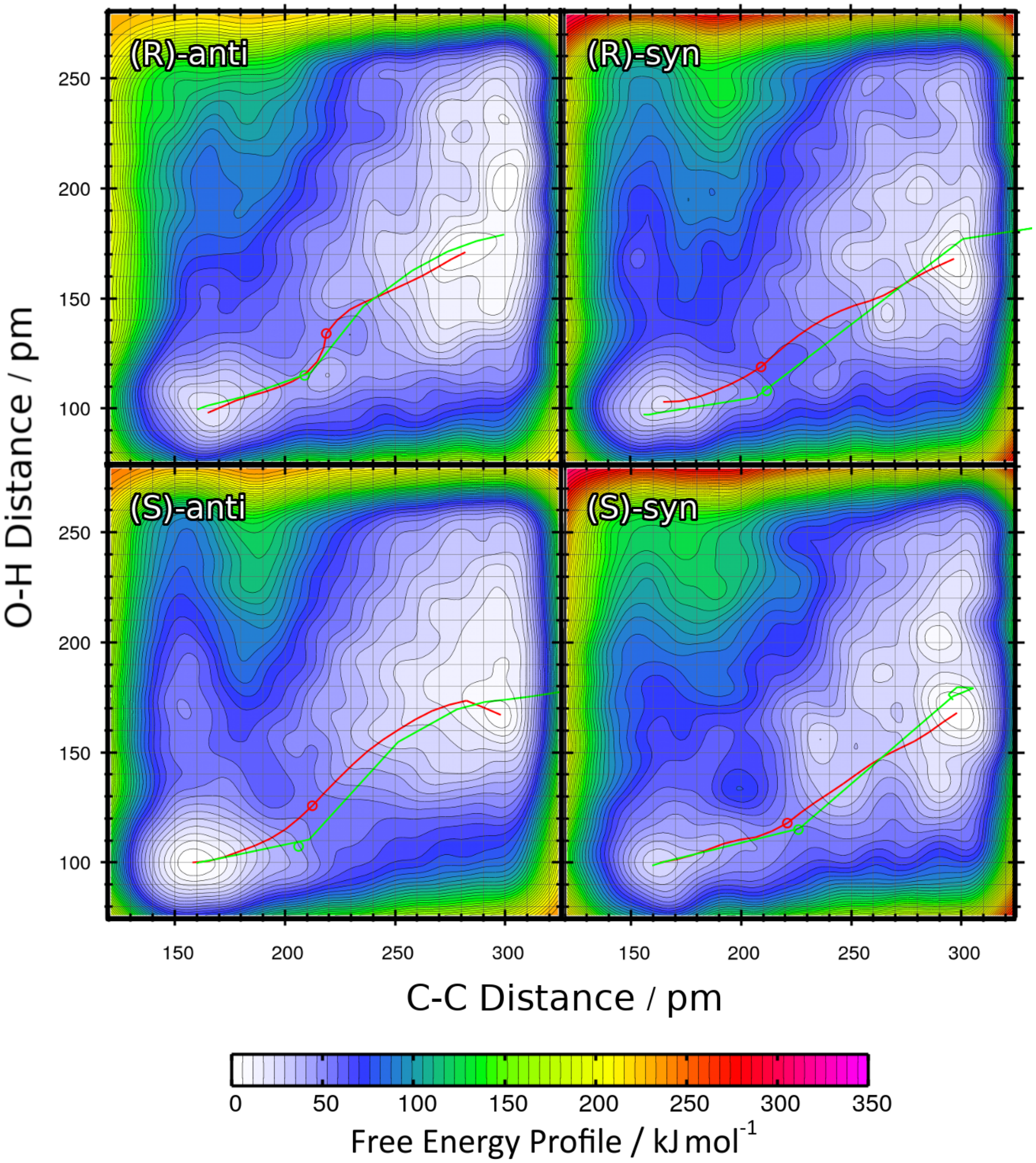

Figure S3: Free energy profiles of the Aldol reactions (R = Et) in vacuum computed from HyAIMD Metadynamics; definition of collective variables see Figure 4. Red curves depict the minimum energy paths from educts *(upper-right basin)* to products *(lower-left basin)*. Green curves show the results of static NEB calculations for comparison. Green circles denote the statically determined transition state.

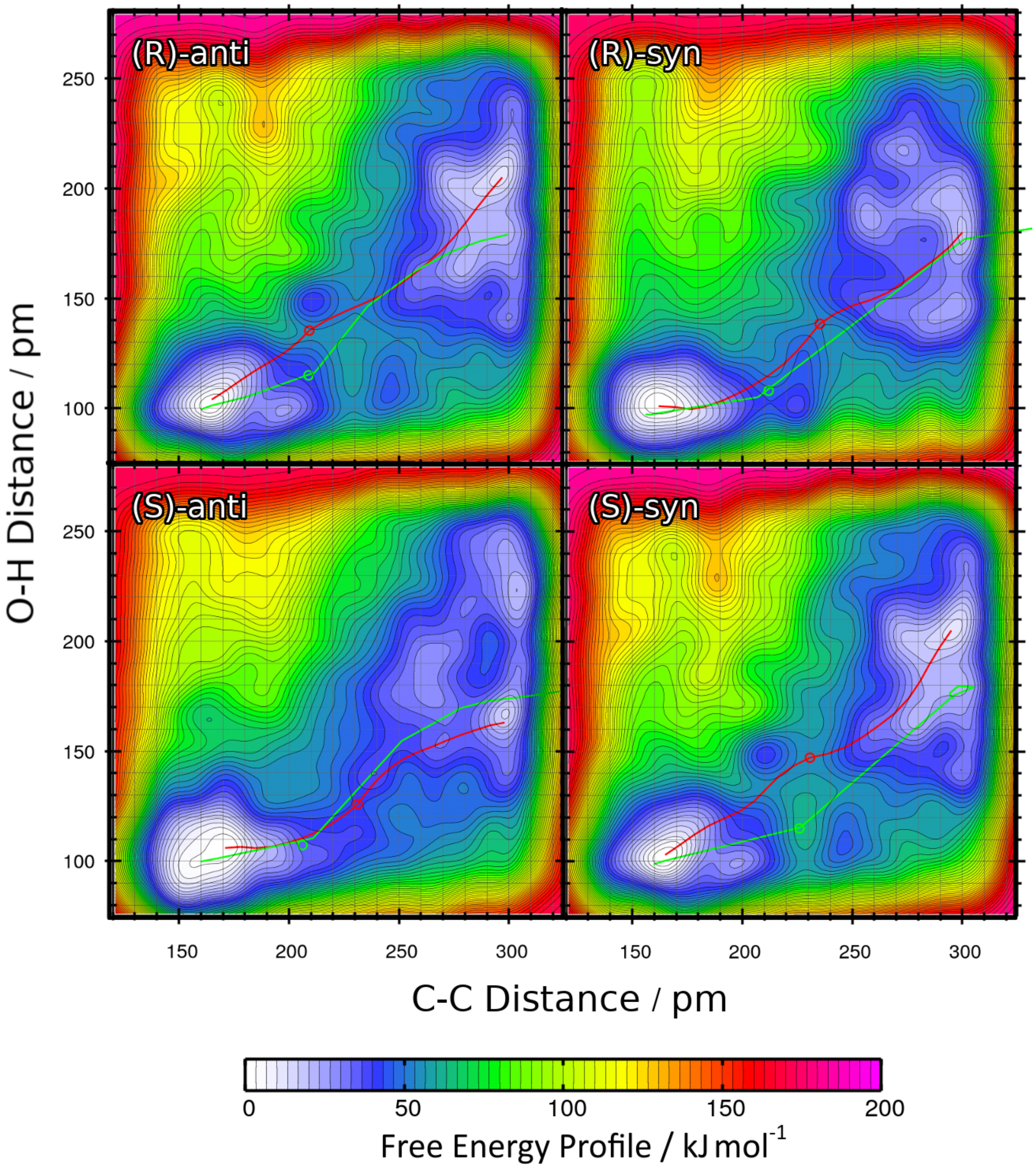

Figure S4: Free energy profiles of the Aldol reactions (R = Et) in DMF computed from HyAIMD Metadynamics; definition of collective variables see Figure 4. Red curves depict the minimum energy paths from educts *(upper-right basin)* to products *(lower-left basin)*. Green curves show the results of static NEB calculations for comparison. Green circles denote the statically determined transition state.

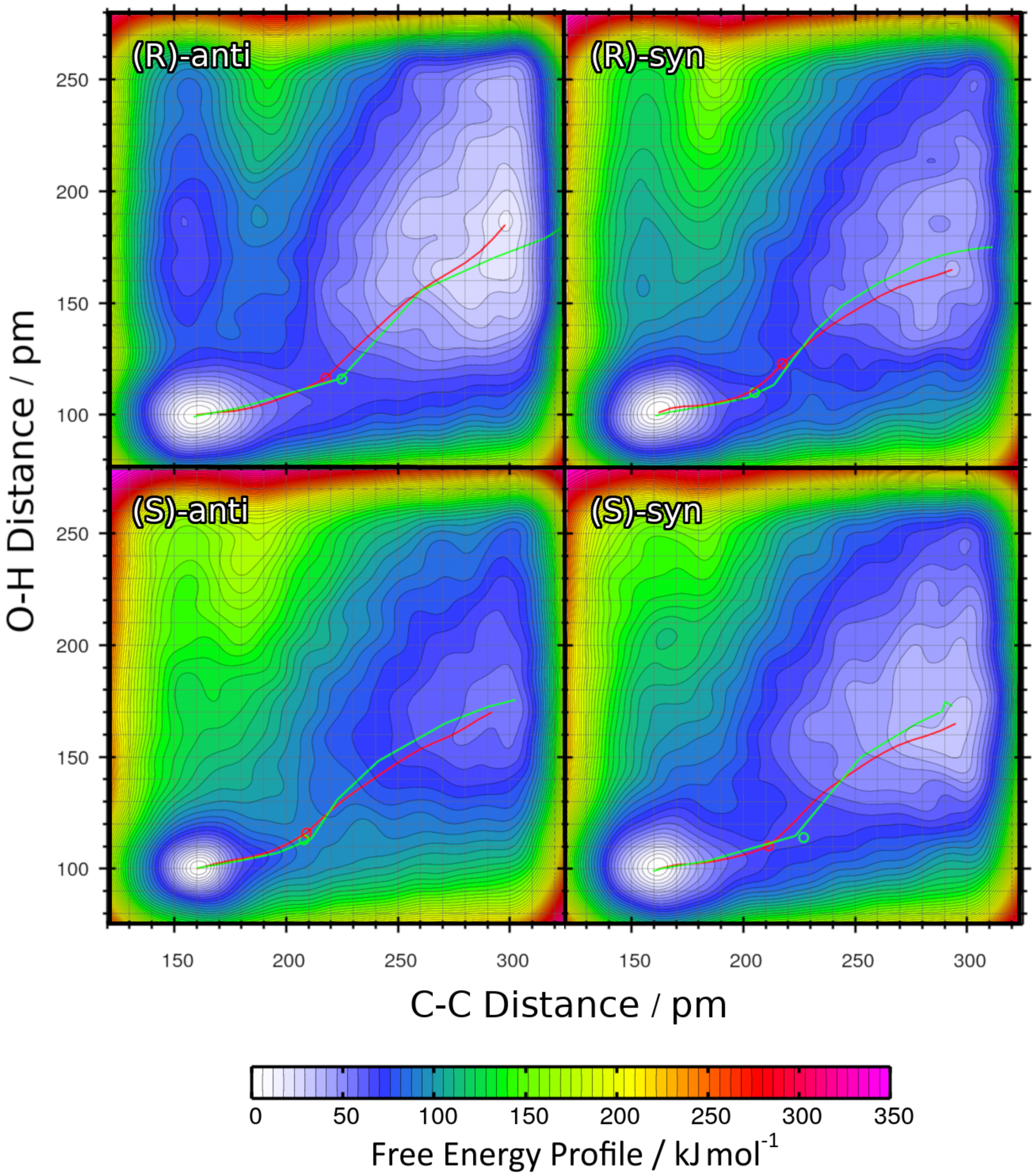

Figure S5: Free energy profiles of the Aldol reactions (R = iPr) in vacuum computed from HyAIMD Metadynamics; definition of collective variables see Figure 4. Red curves depict the minimum energy paths from educts *(upper-right basin)* to products *(lower-left basin)*. Green curves show the results of static NEB calculations for comparison. Green circles denote the statically determined transition state.

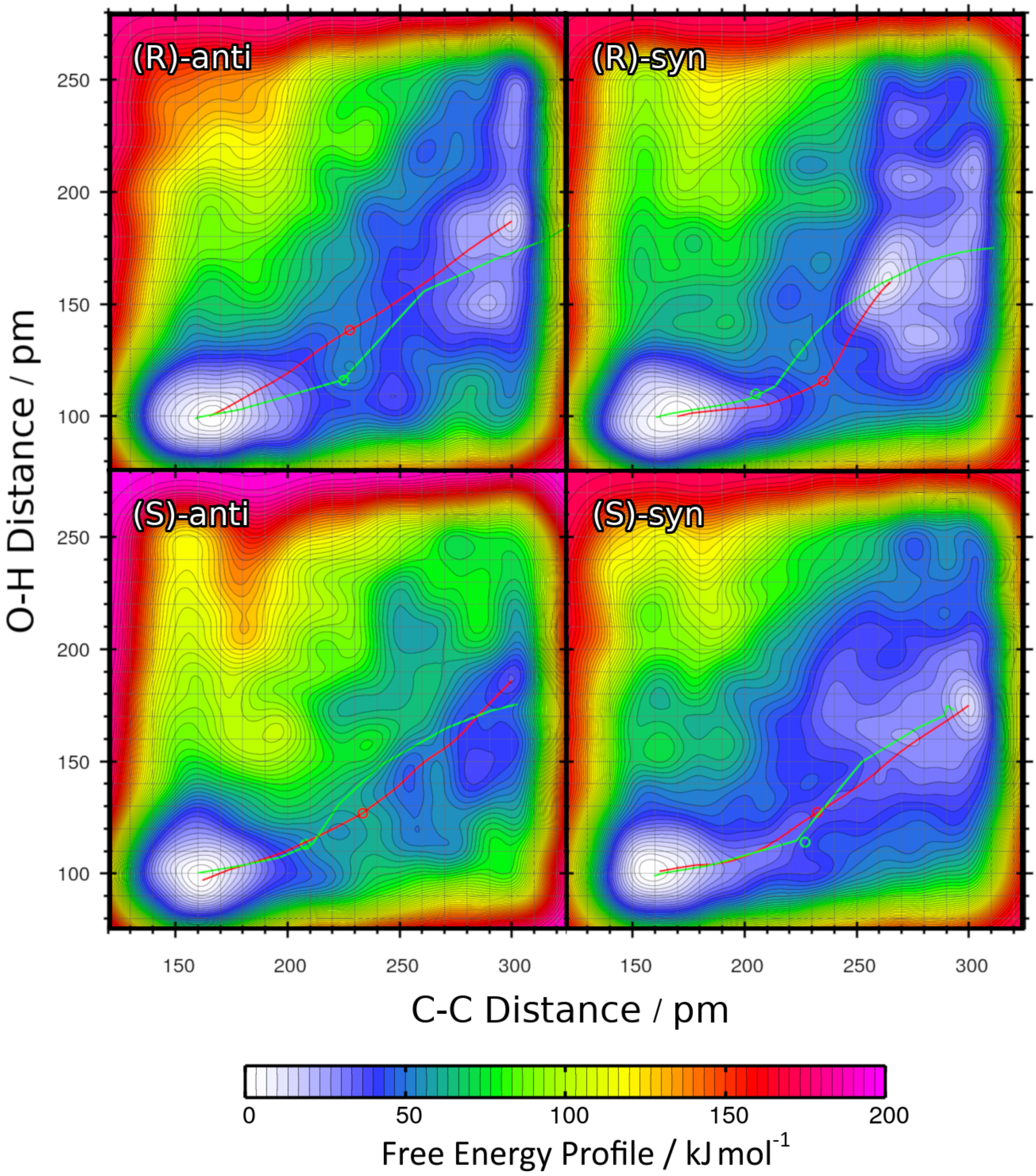

Figure S6: Free energy profiles of the Aldol reactions (R = iPr) in DMF computed from HyAIMD Metadynamics; definition of collective variables see Figure 4. Red curves depict the minimum energy paths from educts *(upper-right basin)* to products *(lower-left basin)*. Green curves show the results of static NEB calculations for comparison. Green circles denote the statically determined transition state.

Minimal Free Energy Pathways from Metadynamics

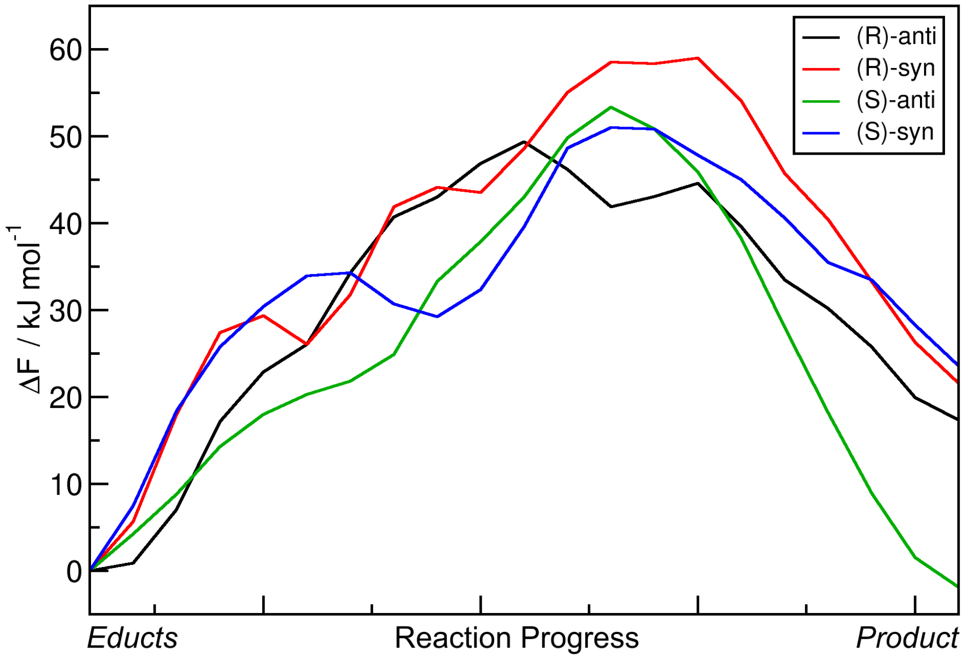

Figure S7: One-dimensional free energy profiles of Aldol reactions (R = Et) in vacuum obtained from HyAIMD Metadynamics; corresponds to red curves in Figure S3.

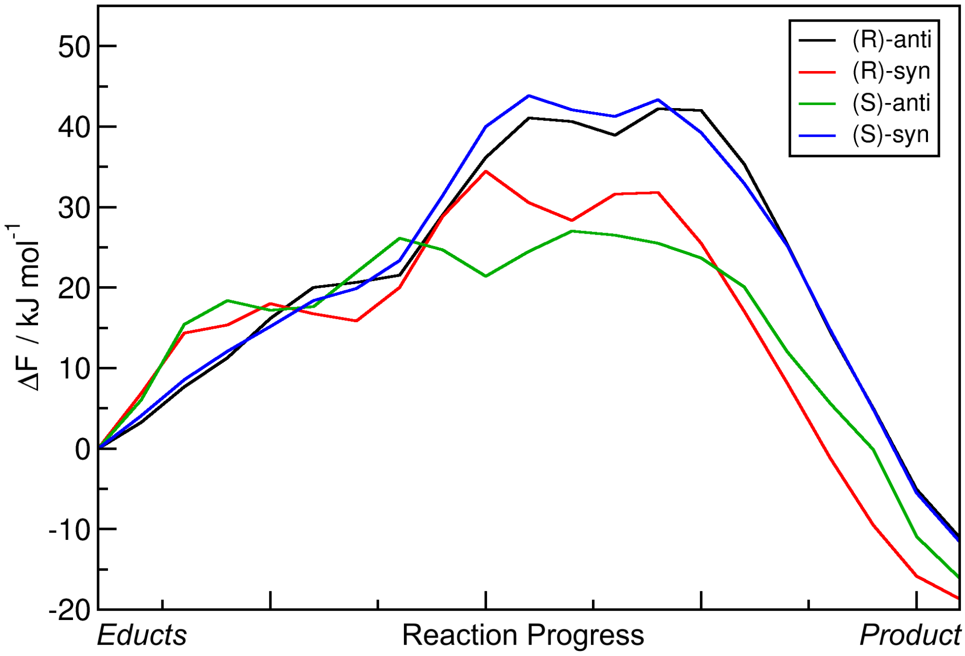

Figure S8: One-dimensional free energy profiles of Aldol reactions (R = Et) in DMF obtained from HyAIMD Metadynamics; corresponds to red curves in Figure S4.

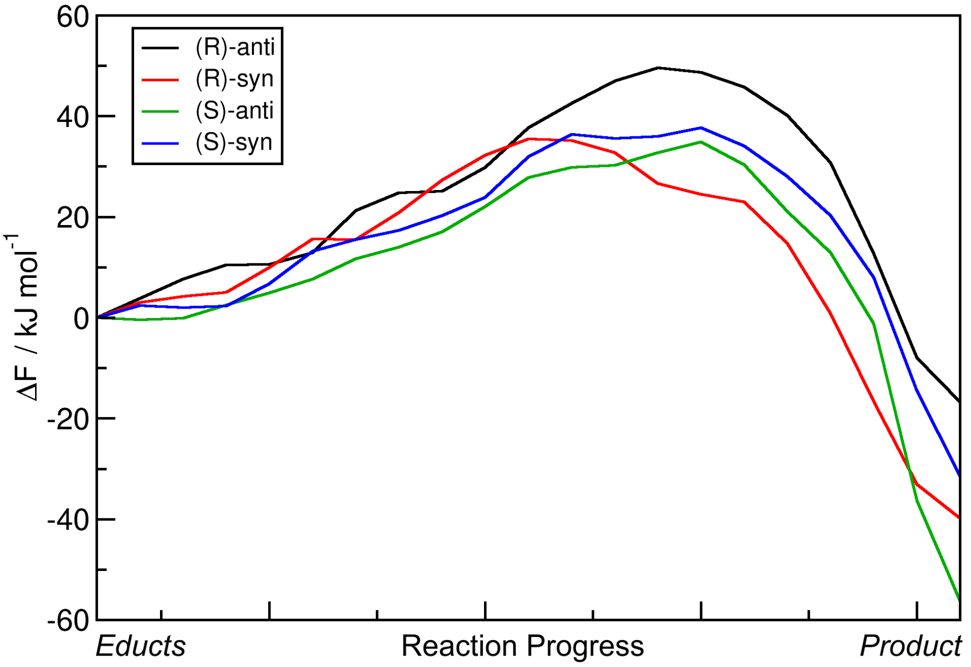

Figure S9: One-dimensional free energy profiles of Aldol reactions (R = iPr) in vacuum obtained from HyAIMD Metadynamics; corresponds to red curves in Figure S5.

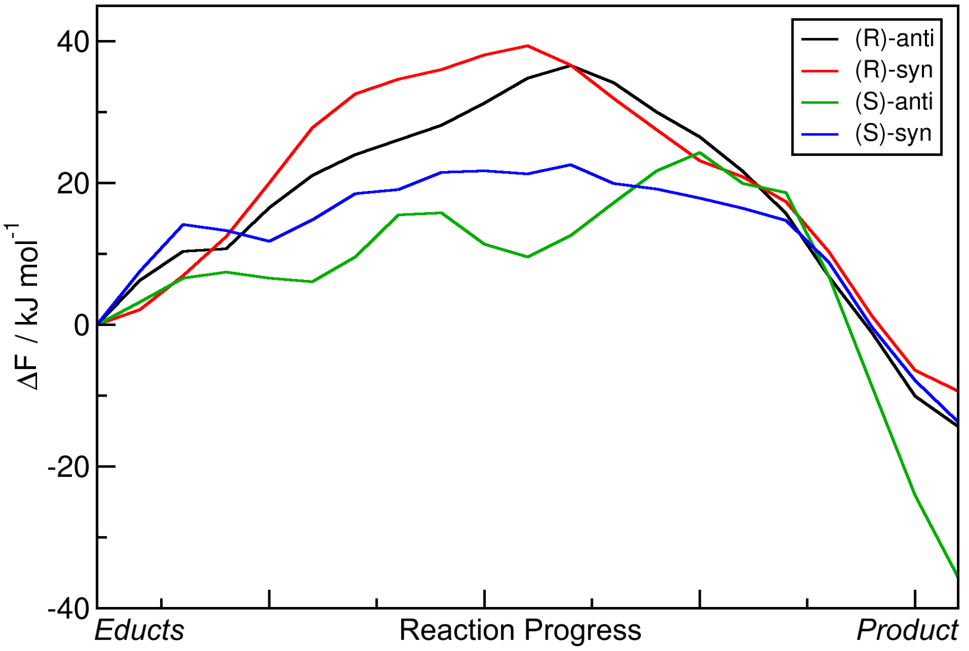

Figure S10: One-dimensional free energy profiles of Aldol reactions (R = iPr) in DMF obtained from HyAIMD Metadynamics; corresponds to red curves in Figure S6.

Reaction Free Energies and Free Energy Barriers

Table S3: Total reaction free energies $\Delta_R F$ and reaction free barriers $\Delta F^{\ddagger}$ for the Aldol reactions (R = Et) in vacuum from HyAIMD Metadynamics; extracted from Figure S7.

**Product**	$\mathbf{\Delta_R F}$ **/ kJ mol**$^{-1}$	$\mathbf{\Delta F^{\ddagger}}$ **/ kJ mol**$^{-1}$
(R)-anti	17.33	49.38
(R)-syn	21.56	59.01
(S)-anti	−1.90	53.36
(S)-syn	23.59	50.99

Table S4: Total reaction free energies $\Delta_R F$ and reaction free barriers $\Delta F^{\ddagger}$ for the Aldol reactions (R = Et) in DMF from HyAIMD Metadynamics; extracted from Figure S8.

**Product**	$\mathbf{\Delta_R F}$ **/ kJ mol**$^{-1}$	$\mathbf{\Delta F^{\ddagger}}$ **/ kJ mol**$^{-1}$
(R)-anti	−11.06	42.24
(R)-syn	−18.66	31.79
(S)-anti	−17.71	25.40
(S)-syn	−11.52	43.84

Table S5: Total reaction free energies $\Delta_R F$ and reaction free barriers $\Delta F^{\ddagger}$ for the Aldol reactions (R = iPr) in vacuum from HyAIMD Metadynamics; extracted from Figure S9.

**Product**	$\mathbf{\Delta_R F}$ **/ kJ mol**$^{-1}$	$\mathbf{\Delta F^{\ddagger}}$ **/ kJ mol**$^{-1}$
(R)-anti	−16.69	49.64
(R)-syn	−39.82	35.51
(S)-anti	−56.30	34.92
(S)-syn	−31.49	36.43

Table S6: Total reaction free energies $\Delta_R F$ and reaction free barriers $\Delta F^{\ddagger}$ for the Aldol reactions (R = iPr) in DMF from HyAIMD Metadynamics; extracted from Figure S10.

**Product**	$\mathbf{\Delta_R F}$ **/ kJ mol**$^{-1}$	$\mathbf{\Delta F^{\ddagger}}$ **/ kJ mol**$^{-1}$
(R)-anti	−14.33	36.54
(R)-syn	−9.35	39.35
(S)-anti	−35.63	24.29
(S)-syn	−13.70	22.59
